# P13 Finding an answer for an adolescent with back pain

**DOI:** 10.1093/rap/rkab068.012

**Published:** 2021-10-19

**Authors:** Sharmin Nizam

**Affiliations:** Mid Yorkshire NHS Trust, Wakefield, United Kingdom

## Abstract

**Case report - Introduction:**

This is the case of an adolescent referred to rheumatology following 5 years of back pain. After years of trying a number of treatments without much success, the cause was found to be a previously undiagnosed urological pathology. The case highlights awareness of non-rheumatological causes and incidental findings which can redirect a patient towards more appropriate treatment and reduce the potential for long-term adverse health issues and anxiety.

**Case report - Case description:**

B was referred age 16 to rheumatology with a 5-year history of lower back pain. She had previously seen paediatricians with symptoms initially attributed to constipation due to intermittent straining and hard stool. However, constipation remedies had not relieved the pain which progressed gradually to a more persistent dull ache with impact on daily activities. Various analgesics (including paracetamol and non-steroidal anti-inflammatories), exercises and acupuncture had not helped. There was no history of recurrent urinary tract infections or symptom correlation with fluid intake, menstruation or bowel habit. No inflammatory features or connective tissue disease symptoms were noted and family history was unremarkable Clinical examination was normal apart from mild tenderness in the lumbar region.

Rheumatoid factor was borderline positive (15 iu/mL) with the rest of blood tests normal including renal function, inflammatory markers (CRP, ESR), anti CCP and ANA. She had minimal microscopic haematuria without proteinuria.

MRI spine in 2015 was normal. In view of her young age and symptoms affecting daily activities, STIR sequence spinal MRI was requested. This excluded any new or old inflammatory changes but incidentally identified a dilated left pelvi-calyceal system.

Renal ultrasound confirmed a grossly hydronephrotic left kidney with hydroureter and minimal renal tissue suggesting longstanding obstruction. No calculi were seen. The patient was referred to urologists. Further investigations (including MRI abdomen) confirmed similar findings and a distal ureteric stricture. A MAG 3 renogram showed a normal right kidney but only 12% functioning of the left kidney.

Urologists have advised surgery (removal of left kidney and ureter) which may relieve symptoms or a conservative non-surgical approach (continue analgesia, physiotherapy and monitoring). The patient and her family are relieved to have a possible cause identified and are considering the surgical option due to ongoing flank discomfort.

**Case report - Discussion:**

This was an interesting finding of hydroureter and hydronephrosis causing longstanding back pain presenting to rheumatologists. Until completion of the spondyloarthropathy protocol MRI (STIR images), aetiology had been unclear.

Hydronephrosis and hydroureter has no specific age or racial predilection. Signs and symptoms may depend on whether obstruction is acute/chronic. Chronic cases may be asymptomatic or present as a dull discomfort (like this case). Some cases may only present in adulthood with pain precipitated by fluid intake.

Blood tests may show impaired kidney function. Post-mortem studies suggest 50% of people have at least one renal abnormality (e.g., renal cysts, duplex ureters) with autopsy series incidence of hydronephrosis reported as 3.1%.

Causes include anatomical abnormalities such as vesico-ureteric reflux, urethral strictures (usually present in childhood), calculi, benign prostatic hyperplasia, or intrapelvic neoplasms, pregnancy and infections (e.g., TB). Sudden onset unilateral renomegaly was reported in one case of primary Sjogren’s with lymphocytic interstitial nephritis and positive Sjogren’s autoantibodies. Our patient has no clinical or serological evidence of connective tissue disease.

Minor pelvi-calyceal distension can occur as a normal finding in well-hydrated patients and pregnancy. However, significant hydronephrosis requires assessment to determine cause as it may affect long term renal function. Imaging via computed tomography, ultrasound and urograms can help guide further management.

In this case the preceding cause and duration of pathology is unknown. Sterile, giant hydronephrosis treatment options include observation and ureteric stent or nephrostomy in patients unfit for surgery. Nephrectomy is advised for pain and recurrent infection in a non-functioning kidney. Complications may include bowel perforation, vascular injury and urine leakage. Both open and minimally invasive procedures have good reported outcomes. The COVID-19 pandemic and exams have affected timing of any elective procedures and the patient understands surgery may or may not offer complete symptom resolution.

**Case report - Key learning points:**

- Non-inflammatory causes of back pain should always be considered in cases of persistent back pain, particularly in young people to ascertain if there is a treatable cause
- Hydronephrosis cases can be asymptomatic or present with vague, intermittent, non-specific abdominal symptoms with normal physical examination with or without haematuria. This can cause diagnostic uncertainty and delay referral to urology and appropriate renal investigations
- Assessment of renal function (including MAG 3 renogram) is important to guide further management
- Surgical interventions (pyeloplasty/nephrectomy) may ease symptoms long term but there is no guarantee of a successful outcome and operative risks need to be considered too
- Left undiagnosed, potentially this patient could have had further disruption to daily activities and both physical and mental well being

